# 368. Impact of Social Determinants of Health (SDOH) on Outcomes of OPAT (Outpatient Parenteral Antimicrobial Therapy)

**DOI:** 10.1093/ofid/ofae631.109

**Published:** 2025-01-29

**Authors:** Mike Sportiello, Colleen Burgoyne, Alexandra Yamshchikov

**Affiliations:** University of Rochester Medical Center, Rochester, NY; Univ. of Rochester, Rochester, New York; University of Rochester School of Medicine and Dentistry, Rochester, New York

## Abstract

**Background:**

Complex infections may warrant extended antibiotics traditionally reserved for inpatient settings. OPAT is safe and cost effective, allowing return to regular activities and decreased risk of hospitalization. Understanding of SDOH-sensitive risk parameters and impact on adverse events is needed to guide safety and adherence interventions.

**Figure 1.**
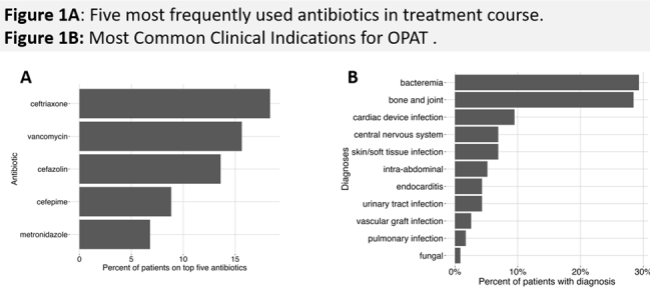
Most Common OPAT Antibiotics and Indications in Study Cohort

**Methods:**

Records of patients treated 7/15-10/31/2023 by URMC OPAT program, exclusive of concurrent Transitions of Care Program, were retrospectively reviewed for race, social vulnerability index, insurance type, substance use, needs insecurity, household composition, and outcomes such as OPAT-related orders errors, intravascular device (IVAD) complications, emergency utilization, organ system adverse events, and clinical outcome. A composite Undesirable OPAT Outcome was derived. The impact of SDOH was assessed by chi-square, univariate and multivariate logistic regression using R v4.3 3, rstatix v0.7.2, and sjPlotv2.8.15.

**Table 1.**
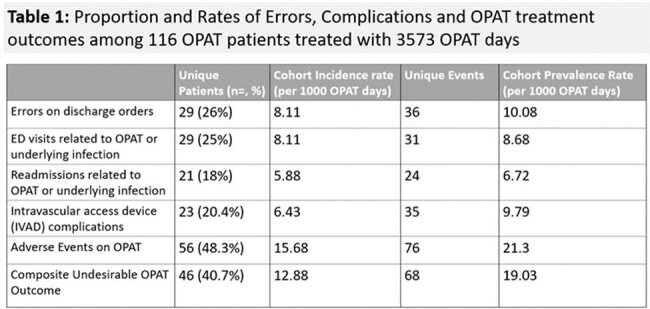
Proportion and Rates of Errors, Complications, and OPAT Treatment Outcomes in Study Cohort

**Results:**

Total 116 patients received 3573 OPAT days for varying indications (Fig 1). High rates of discharge order errors, ED utilization, IVAD complications, adverse events, and composite Undesirable Outcomes were noted (Table 1). Those with public insurance (OR=13.6, CI: 1.95-322) or who used cannabis (OR=5.23 CI: 1.1- 27.2) were more likely to incur a laboratory order error on discharge. Patients of color were more likely to experience IVAD complications (OR=3.61, CI: 1.1-12.6) and multiple organ system adverse events (OR= 6.55, CI: 1.45-34.9). High-risk tobacco use was associated with shortened OPAT duration due to adverse event (OR=28.47, CI: 1.72-1299) (Fig 2). OPAT patients with public insurance were least likely to follow up with the ID clinic (OR=7.65, CI: 1.27-152). Time to ID follow-up correlated with multiple SDOH parameters, with publicly insured and persons of color experiencing significantly longer time to follow-up appointments (Fig 3). Incurring a non-IVAD adverse event correlated to composite undesirable outcome in multivariate analysis.

**Figure 2.**
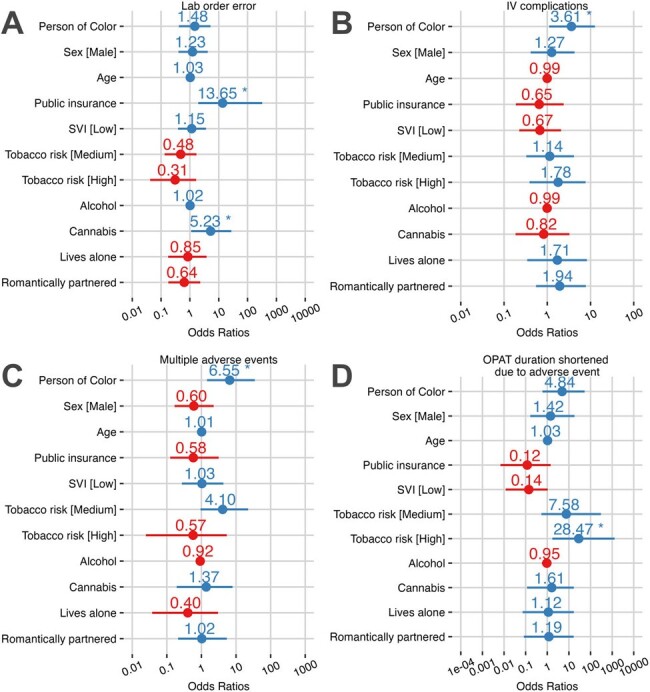
Odds Ratios in Univariate Analysis of OPAT Complications Based on Social Determinants of Health

**Conclusion:**

Social determinants may be predictors in OPAT-related unwanted events. A dedicated focus of evaluating SDOH as contributing drivers of adverse OPAT outcomes may assist creating interventions to support marginalized patient groups.

**Figure 3:**
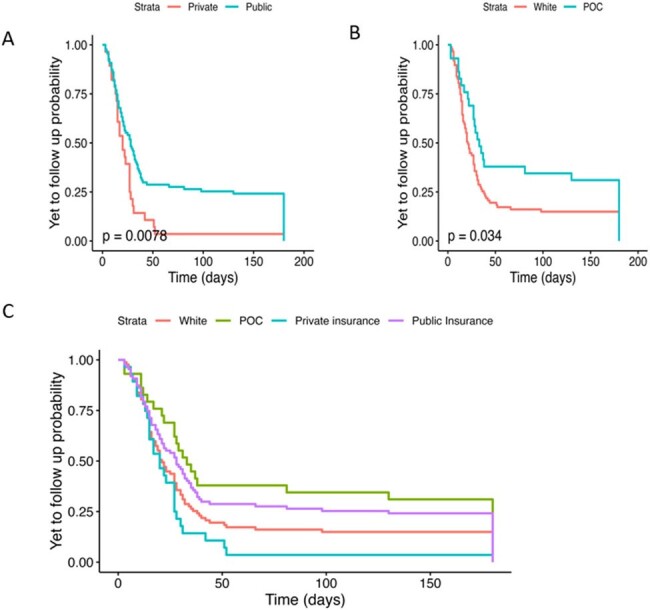
Time-to-event probability analysis for timing of post-discharge ID clinic follow up among OPAT patients by Insurance Type (3A), Race (3B), and All Patients (3C)

**Disclosures:**

**All Authors**: No reported disclosures

